# Circadian Regulation in Diurnal Mammals: Neural Mechanisms and Implications in Translational Research

**DOI:** 10.3390/biology13120958

**Published:** 2024-11-22

**Authors:** Yirun Jiang, Jiaming Shi, Jun Tai, Lily Yan

**Affiliations:** 1 Department of Otolaryngology, Head and Neck Surgery, Capital Institute of Pediatrics, Beijing 100020, China; jiang.yirun@sohu.com (Y.J.); trenttj@163.com (J.T.); 2 Department of Psychology, Michigan State University, East Lansing, MI 48824, USA; shijiami@msu.edu; 3 Neuroscience Program, Interdisciplinary Science & Technology Building (ISTB), Michigan State University, East Lansing, MI 48824, USA

**Keywords:** circadian clock, light, melatonin, familial natural short sleep, sleep apnea, Smith–Magenis syndrome

## Abstract

Diurnal and nocturnal mammals have evolved unique behavioral and physiological adaptations to optimize survival for their day- or night-active lifestyle. The mechanisms underlying the opposite activity patterns are not fully understood but likely involve the interplay between the circadian time-keeping system and various arousal- or sleep-promoting factors, e.g., light or melatonin. Although the circadian systems between the two chronotypes share considerable similarities, the phase relationships between the principal and subordinate oscillators are chronotype-specific. While light promotes arousal and wakefulness in diurnal species like us, it induces sleep in nocturnal ones. Similarly, melatonin, the hormone of darkness, is commonly used as a hypnotic in humans but is secreted in the active phase of nocturnal animals. Thus, the difference between the two chronotypes is more complex than a simple reversal, as the physiological and neurological processes in diurnal mammals during the day are not equivalent to that of nocturnal ones at night. Such chronotype differences could present a significant translational gap when applying research findings obtained from nocturnal rodents to diurnal humans. The potential advantages of diurnal models are being discussed in a few sleep-related conditions including familial natural short sleep (FNSS), obstructive sleep apnea (OSA), and Smith–Magenis syndrome (SMS). Considering the difference in chronotype, a diurnal model will be more adequate for revealing the physiology and physiopathology pertaining to human health and disease, especially in conditions in which circadian rhythm disruption, altered photic response, or melatonin secretion is involved. We hope the recent advances in gene editing in diurnal rodents will promote greater utility of the diurnal models in basic and translational research.

## Simple Summary

While humans are diurnal, commonly used laboratory rodents are nocturnal, i.e., sleeping mostly during the day and being active at night. The unique day- or night-active lifestyle has been shaped by their evolutionary history and likely involves the interplay between the circadian time-keeping system and various arousal- or sleep-promoting factors, e.g., light or melatonin. In the present review, we first discuss the similarities and differences in these regulatory processes between the two chronotypes and the potential mechanisms that could have contributed to the diurnality–nocturnality switch. Next, using a few sleep-related conditions as examples, we discuss the advantages of using diurnal rodent models in translational research, especially under conditions in which circadian rhythm disruption, altered photic response, or melatonin secretion is involved.

## 1. Introduction

The daily light/dark (LD) cycle is the most salient environmental factor influencing the temporal organization of behavior and physiology in living organisms [1]. There are several temporal patterns (chronotypes) based on the phase of the LD cycle in which the animals are most active, i.e., nocturnal, diurnal, crepuscular (active at dawn and dusk), or cathemeral (active in both day and night). Humans are diurnal, as we sleep at night and are active during the day. Even for those so-called “night owls” or “morning larks”, who prefer to stay up until late at night or wake up in the early morning, their active/rest rhythm is still predominantly diurnal. Among modern mammals, only ~20% are diurnal like humans, nearly 70% are nocturnal, and 10% are crepuscular or cathemeral [2]. Diurnal and nocturnal mammals have been set apart through a series of divergent evolutionary pathways over a period of ~200 million years, which has equipped them with a range of unique behavioral and physiological adaptations to optimize survival for their day- or night-active lifestyle [3,4,5,6]. Daily rhythms in behavior and physiology are directly regulated by an internal time-keeping system called the circadian clock and by environmental factors including the light/dark condition. The similarities and differences in the regulatory processes between chronotypes will be discussed in this review. Additionally, since the predominant animal model in translational research is nocturnal mice, the potential factors that may present as a translational limitation and how to mitigate this by a diurnal model will also be discussed, using a few sleep-related conditions as examples.

## 2. Adaptive Value of the Circadian System

The daily patterns of diurnal and nocturnal animals are regulated by an internal time-keeping system, the circadian clock, which anticipates daily fluctuations in the environment, e.g., light/dark, temperature, food availability, predator activities, etc., and coordinates the temporal organization of behaviors and physiology to align with the changing environment [7,8]. The leading theory for the evolution of the circadian clock is the “escape from light” hypothesis, which suggests that in single-cell organisms, the circadian clock evolved to time-crucial biochemical events to avoid the harmful effects of light, particularly UV light [9,10]. The feature of the circadian clock is preserved through evolution, which is hypothesized to be adaptive in the cyclic environment on our planet. The adaptive advantage of a precise clock has been supported by experiments performed in various model organisms, including cyanobacteria, flies, and rodents [11,12]. In cyanobacteria, when various strains with faster or slower clocks (shorter or longer circadian period) were cultured together in different periods of LD cycles, the strain whose clock period closely matched to the LD cycle always out-competed other strains whose clock periods were more deviated from the LD cycle [13]. In flies with an intact clock with a circadian period near 24 h, their life span was greater in a 24 h than in non-24 h LD cycle [14,15]. In rodents, it has been shown that a faster clock or shorter period is selected against in terms of both survival and reproduction in mice living in a semi-natural enclosure [16], leading to heart and renal pathology in hamsters in a laboratory study [17]. In humans, the health impact of a disturbed circadian system has been well documented in shift workers, who are at higher risk for having both physical and mental health issues [18].

## 3. Regulatory Mechanisms of the Circadian System

The internal time-keeping circadian system coordinates the temporal organizations of molecular, cellular, and physiological processes across the body and ensures the timing of behavior and physiology, e.g., sleep and wakefulness are adapted to the environmental day–night cycle [19]. In mammals, the circadian system is organized in a hierarchical manner, with the principal brain clock in the hypothalamic suprachiasmatic nucleus (SCN) on top of the hierarchy, coordinating the subordinate clocks in extra-SCN brain regions and in peripheral tissues and organs [20].

### 3.1. Molecular Machinery

The circadian clock has a molecular basis that has been described as interlocked transcriptional/translational feedback loops (TTFLs), which is conserved to a great extent in various model organisms from fungi and fruit flies to mammalian species [21,22,23]. The details in the molecular machinery have been extensively reviewed and recently updated [24,25,26,27,28] and thus will only be briefly discussed here. In mammals, the molecular machineries or TTFLs are constituted by a set of “clock genes” and their protein products. In the primary TTFL, the transcription factors CLOCK and BMAL1 activate the transcription of their negative regulators *PER1/2* and *CRY1/2* in a ~24 h cycle. This core oscillation is further stabilized by auxiliary feedback loops, in which TTFL-driven expression of *REV-ERB* and *RORA* control the transcription of *BMAL1*. Although the model of the mammalian circadian clock was built initially based on work in laboratory mice, subsequent findings in diurnal rodents [29,30], sheep [31], and humans [32,33] confirmed that the same mechanisms are conserved between diurnal and nocturnal mammals. The molecular machinery exists in vivo within individual cells of almost all tissues/organs and in vitro in cultured cell lines [27,34,35]. This core molecular clock drives the circadian oscillation of downstream genes, which are also referred to as clock-controlled genes [19]. Recent transcriptome studies using all major organs in nocturnal rodents and diurnal non-human primates have revealed that 45–80% of protein-coding genes show circadian expression in at least one organ [36,37].

### 3.2. Phase Relationship Between SCN, Extra-SCN Brain Regions, and Peripheral Tissues/Organs

SCN: Although the regulatory mechanisms of the molecular clocks are essentially the same regardless of the animals being diurnal or nocturnal, chronotype-specific phase relationships have been documented. In the SCN, expressions of many clock genes and clock-controlled genes have been found to oscillate in similar phase relative to the LD cycle in diurnal and nocturnal mammals [38]. Additionally, the overall electrical activity and metabolism within the SCN are in phase between diurnal and nocturnal rodents, that is, high during the day and low at night [38]. These findings suggest that the SCN serves as a circadian timekeeper by keeping track of the environmental LD cycle, but it does not dictate the phase in which an animal is more active or sleepy.

Extra-SCN and periphery: In contrast to the same temporal pattern in gene expression, electrical activity, and metabolism within the SCN, other brain regions and peripheral tissues/organs show complex differences between diurnal and nocturnal mammals [38]. In many brain regions and peripheral tissues/organs, the expression of clock genes or their protein products often exhibits an anti-phase relationship between diurnal and nocturnal species, corresponding to their behavioral and physiological rhythms [39,40,41,42,43]. However, the circadian oscillations in those subordinate clocks between the two chronotypes are more complex than a simple phase reversal. In diurnal Nile grass rats, it has been found that the expressions of PER2 are only a few hours apart in some regions and similarly phased in other regions compared to nocturnal laboratory rats [44,45,46]. Consistent results have been found in transcriptomic studies comparing the data from a diurnal non-human primate, the olive baboon (*Papio anubis*), and nocturnal laboratory mice, which showed that while the peak expression of core clock genes was in phase within the SCN, it was ~12 h out of phase in many other brain regions or peripheral tissues, or shifted by 6–15 h in others depending on tissue types [36,37]. These findings collectively support the view that there is no single simple switch that causes some animals to be nocturnal and others to be diurnal [47].

## 4. Mechanisms Underlying the Chronotype Switch

The mechanisms underlying the switch between a diurnal vs. nocturnal behavioral pattern are not well understood and likely involve complex neural- and gene-regulatory networks. We will focus our discussion on the structural and functional differences in local circuitry downstream of the SCN and the distinct response to external (light) or internal cues (melatonin) between the two chronotypes.

### 4.1. Structural and Functional Differences in Local Circuitry

To explore the mechanisms underlying the switch between diurnal vs. nocturnal rhythms, the neural pathway regulating the daily rhythm of glucocorticoids from the SCN neurons containing vasopressin to the paraventricular nucleus (PVN) was examined [48]. The infusion of vasopressin into the PVN promoted adrenal glucocorticoid release in diurnal Sudanian grass rats (*Arvicanthis ansorgei*) but inhibited the glucocorticoid release in nocturnal rats. The opposite response to vasopressin led to the hypothesis that a difference in the local circuit of the PVN involving either glutamatergic or GABAergic neurons contributes to the reversal of daily rhythms in glucocorticoid secretion. This hypothesis was supported by the different distribution of glutamatergic and GABAergic neurons in diurnal Nile grass rats (*Arvicanthis niloticus*) and nocturnal Norway rats [49]. Specifically, in the lateral habenula, a cluster of GABAergic neurons was present in diurnal but completely absent in nocturnal rats. The lateral habenula is involved in regulating circadian rhythms and sleep, revealed by studies lesioning/transecting the fasciculus retroflexus, the major efferent projection from the nucleus. The procedure resulted in an altered temporal organization of locomotor activity in Syrian hamsters [50] and REM sleep disturbances in laboratory rats [51,52]. Using a transcriptomic approach, a recent study examined 17 brain regions in CBA/CaJ mice following a behavioral nocturnal–diurnal switch in their wheel-running activity, in which the habenula emerged as the most affected region [53]. Although the reward-based behavioral plasticity is likely not the same as the nocturnal–diurnal switch between species shaped by their evolutionary history, the results from the transcriptome study suggest the habenula could be a potential “hotspot” underlying the switch. Therefore, the presence or absence of GABAergic neurons in local circuits within the habenula may contribute to the reversal in the temporal pattern of locomotor activity and sleep in diurnal rodents compared to nocturnal ones.

In addition to a possible local on-off switch through GABAergic or glutamatergic neurons, functional differences in systems directly regulating sleep/arousal likely contribute to the diurnal or noctural temporal pattern. A likely candidate is the hypothalamic hypocretin/orexin system, which has been implicated in many important physiological functions, including promoting wakefulness and arousal [54,55]. The orexin peptides are well conserved across mammals including mice, rats, dogs, pigs, and humans [56]. Orexin-containing neurons are localized in the lateral hypothalamus in both diurnal and nocturnal rodents [57,58], as well as in humans [59,60]. In diurnal rodents, orexin neurons are activated by light, an arousal cue for diurnal species [61]; in nocturnal rodents, these neurons are activated by darkness [62]. The differential response to light may be related to differences in the anatomic distribution of orexin receptors. Orexin peptides bind to two types of G-protein-coupled receptors, type 1 (OX1R) and type 2 (OX2R) [63]. Although the distribution of these receptors is generally similar, species-specific expression has been found such that OX1R mRNA was detected in the caudate putamen and ventral tuberomammillary nucleus in diurnal grass rats but not in nocturnal mice [64].

Using a hypothesis-driven approach, these previous studies have helped to reveal several differences in neural networks corresponding to the temporal pattern in each chronotype. However, we do not know whether those systems constitute all of the major players or if they are only part of the mechanisms underlying the switch between chronotypes. It is possible they are just a few candidates in a much larger constellation of factors collectively contributing to the adaptation from a nocturnal to diurnal lifestyle or vice versa. Therefore, in addition to the hypothesis-driven examinations that focus on specific candidates based on prior knowledge, an unbiased analysis such as a transcriptomic and whole-brain spatial atlas assay could be an effective approach to reveal novel pathways that have likely been missed by the previous candidate-based approach [65]. The chronotype- or species-specific patterns will help identify possible players as the first step, and follow-up functional studies will be essential for establishing the role played by each player.

### 4.2. Distinct Response to Light

While ambient light can entrain the circadian clock in a similar way between diurnal and nocturnal species, light can also directly influence behavior independent from the circadian drive in a chronotype-specific way. In diurnal mammals like humans, light enhances activity and promotes wakefulness, while in nocturnal rodents, e.g., mice or rats commonly used in laboratory research, light inhibits activity and induces sleep [66,67].

Light is first processed by the retina. Many features of the retina are specialized for operating in either daylight or night hours, e.g., 30–40% of photoreceptors are cones (cone-rich) in diurnal rodents vs. ≤3% are cones in nocturnal rodents [68]. Other adaptations in the visual system are also documented, including a relatively larger size of visual regions in the brain of diurnal rodents compared to nocturnal rodents [69]. Beyond these chronotype differences in both diurnal and nocturnal mammals, the non-image-forming effects of light are mediated by intrinsically photosensitive retinal ganglion cells (ipRGCs) containing melanopsin as a photopigment [70,71]. It has been shown that ipRGCs and the classical rod/cone photoreceptors all contribute to circadian photoentrainment in nocturnal rodents [72]. Moreover, melanopsin mediates the light-induced suppression of locomotor activity in nocturnal rodents [73]. The distribution, morphological types, photoresponses, and projections of melanopsin-expressing ipRGCs have been found to be generally similar between diurnal and nocturnal rodents [74,75]. This raises the following intriguing question: what are the species or chronotype differences in the underlying mechanisms that ultimately give rise to the opposite effects of light on behavior?

The divergent behavioral responses are likely due to structural and/or factional differences in direct or indirect retinorecipient brain regions involved in regulating locomotor activity and/or sleep/wake regulation. In the retinorecipient regions, such as the intergeniculate leaflet (IGL) and the olivary pretectal nucleus (OPT), light pulse induced neuronal activation, indicated by an increased cFOS in diurnal grass rats, but it had no effect in the IGL and the opposite response in the OPT in mice [76]. Follow-up lesion studies further support the role of the IGL and OPT in mediating the direct behavioral responses to light or dark in diurnal rodents [77,78,79].

### 4.3. Melatonin

Melatonin is an endogenous factor closely related to circadian and sleep regulation, which has distinct effects in diurnal and nocturnal mammals. The secretion of this hormone is regulated by a multisynaptic pathway from the SCN to the pineal gland [80]. As the hormone of darkness, melatonin is elevated at night but almost absent during the day regardless of the chronotype, as the result of the circadian drive and light-induced suppression [81,82].

The modulatory effects of melatonin on the circadian system have been studied extensively in nocturnal mammals and in humans [83]. In humans, melatonin is often used for preventing or treating the adverse effects associated with jet lag and circadian sleep disorders, e.g., non-24 h sleep–wake syndrome and advanced or delayed sleep phase syndrome [84]. Indeed, in both diurnal and nocturnal rodents, it has been shown that melatonin given prior to or after the dark phase can either advance or delay their circadian rhythms [85,86,87]. There is a substantial body of work characterizing the effects of melatonin on basic circadian processes in humans, which collectively suggest that the response of our circadian system to melatonin administration is similar to that observed in diurnal or nocturnal rodents [88].

In contrast to the rather consistent effects on the circadian system, the effects of melatonin on sleep/arousal are drastically different, evidenced by the fact that melatonin is high in the time that sleep propensity is highest in diurnal mammals but lowest in nocturnal ones. It is also noteworthy that major mouse strains, including C57BL/6, do not synthesize melatonin within the pineal gland due to melatonin synthesis enzyme deficiency [89,90]. Melatonin supplements are a popular over-the-counter sleep aid, supported by the soporific effects observed in studies of human subjects treated with melatonin [91,92,93,94]. Taken together, the evidence supports that melatonin can facilitate sleep directly in a manner that changes across the circadian cycle in humans. These findings have led to the development of several melatonin-based therapeutic agents, e.g., a slow-release melatonin preparation and synthetic melatonin receptor agonists that have been used for the treatment of circadian sleep–wake rhythm disorders that often occur in jet lag, shift work, and certain types of insomnia [95,96]. Altogether, it is well established that melatonin plays an important role in sleep regulation in humans. To better understand melatonin biology as a sleep aid, sleep regulation, and sleep disorders in general, a diurnal model with melatonin secreted in their sleep phase will be more adequate compared to nocturnal ones with melatonin secreted in their active phase.

## 5. Diurnal Models in Studying Sleep and Sleep Disorders

Sleep or sleep-like behavior is ubiquitous across animal species. Sleep is essential to physical and mental health, but many aspects of modern lifestyles induce sleep deprivation. In a 2019 poll of US adults conducted by the National Sleep Foundation, more than 80% reported inadequate sleep at least once per week and 50% reported excessive daytime fatigue [97]. It is also estimated that 60–70% of US middle school and high school students do not receive adequate sleep [98], which coincides with the transient delayed sleep phase observed during adolescence [99]. Chronic sleep deprivation has well-known negative health consequences ranging from cognition to mood to neurodegeneration [100]. Although the characteristics of sleep are diverse across species, ranging from polyphasic with frequent napping in rodents to monophasic consolidated sleep in humans, the fundamental framework involving the circadian and homeostatic drives, as well as the neural circuitry regulating sleep/wake states, are well conserved in mammalian species [101]. Nocturnal mice or rats, are commonly used in sleep studies, which have provided valuable insights for understanding the molecular and neural mechanisms underlying sleep regulation and dysregulation (reviewed in [102,103,104,105,106]). However, given the role of light and melatonin in promoting arousal or sleep in humans, the distinct responses to these factors between nocturnal and diurnal species discussed above likely present a translational gap from studies in mice or rats to sleep-related problems in humans. Next, we discuss a few sleep traits and sleep-related conditions in which a diurnal model could be advantageous for revealing the underlying physiology or pathology.

### 5.1. Familial Natural Short Sleep

Familial natural short sleep (FNSS) is a rare hereditary sleep trait of interest not only to sleep researchers but also to the public. Contrary to the typical 7–8 h of sleep, the FNSS individuals can thrive with only sleeping for 4–6 h per night. Recent research has identified a few genetic mutations in humans that lead to a lifetime reduction in nightly sleep duration [107,108,109,110,111,112]. Though they sleep only 4–6 h at night, FNSS individuals do not suffer from any of the adverse health effects typically associated with sleep deprivation. Compared to unaffected family members, FNSS individuals have less daytime sleepiness, greater resilience to stress, and fewer depression symptoms [113]. Additionally, a mouse model carrying the same mutation is resistant to Alzheimer’s pathology [114]. Whether being able to sleep less is truly a “gift” might be debatable; however, understanding the mechanisms that allow FNSS individuals to maintain good health with less sleep is of great significance, particularly given implications for mental health in the often sleep-deprived modern world.

Several mouse models have been created to decode the secret of the seemingly superior brains of FNSS individuals, but the short sleep phenotype is not as pronounced in mice as that seen in humans. Specifically, mouse models carrying the same human FNSS mutations showed only a subtle (up to 6%) decrease or no effects in the amount of sleep, especially in the light phase when mice are predominantly sleeping. The only mouse FNSS model that had a light-phase short sleep phenotype was *Dec2-P384R*, in which NREM was ~6% less and REM was ~2% less than wild-type mice. This is in contrast to humans with the same mutation, who have almost a 30% reduction in total sleep [110], and contrasts with another *Dec2* human variant (Y362H) associated with a ~20% REM reduction [115]. Mouse lines carrying other human FNSS mutations, i.e., ADRB1, mGluR1, or NPSR1, had an equivalent total sleep time in the light phase as wild-type controls [107,111,112].

Naturally, these observations raise the question regarding the contributing factors for the different sleep phenotype between mouse and humans resulting from the same FNSS mutations. The polyphasic vs. monophasic nature of sleep between rodents and primates could be one of the potential factors. Alternatively, although not mutually exclusively, the opposite chronotype is another likely contributor for the different sleep phenotype between humans and mice. Differences in sleep phenotype could be due to chronotype-specific circadian modulation, such that the FNSS mutation reduces the need for sleep in the resting phase of diurnal species (nighttime in humans) but has no such effect in nocturnal species or only reduces sleep need or napping during their active phase. Another possibility is that the FNSS mutations reduce sleep need in mice during their inactive/sleep phase, but this effect is outpowered or masked by the sleep-promoting effects of light in nocturnal mice. These hypotheses can be tested in a diurnal rodent carrying the FNSS mutation. A diurnal model sharing the same daily temporal pattern of sleep/wake, i.e., active during the day inactive/sleep at night as in humans, is likely to provide valuable insights into how FNSS mutations enabled the brains to achieve superior functions with less sleep.

### 5.2. Obstructive Sleep Apnea

While FNSS is a sleep trait associated with positive physiological and psychological outcomes, most other sleep conditions link to negative health consequences. Obstructive sleep apnea (OSA) is a sleep-related breathing disorder characterized by an abnormal breathing pattern during sleep due to upper airway obstruction [116]. OSA is the most common sleep disorder, with moderate-to-severe OSA estimated to affect 17% of men and 9% of women over 50 in the United States [117]. Patients of OSA experience sleep disturbances including frequent night waking and excessive daytime sleepiness/fatigue, and they have high comorbidity with cardiovascular and metabolic diseases [118].

The mechanisms for the pathogenesis of OSA-associated comorbidities are not fully understood. In addition to the general recognized inflammation, oxidation stress, or sustained sympathetic activation, circadian disruption has been put forth as one of the contributing factors [119,120]. The circadian system appears particularly relevant, given that the hypoxia-inducible factor (IHF), which is involved in the homeostatic regulation of cell and organ functions in response to low oxygen levels, can also regulate circadian rhythms through interacting with the molecular clock [121]. When mice were exposed to an acute 4 h intermittent hypoxia during either the light or dark phase, tissue-specific and phase-dependent transcriptional responses were observed, which persisted in constant dark conditions but largely attenuated in mice deficient of clock genes *Per1* and *Per2*. These findings indicate that the circadian clock or clock genes are involved in mediating tissue-specific transcriptional responses to hypoxia [122]. Another study examined circadian rhythms of the transcriptome in different mouse tissues following 7 days of intermittent hypoxia, which revealed time- and tissue-specific transcriptomic changes, in which cardiopulmonary tissues were more affected than other tissues examined [123]. Although the multi-tissue analysis is not feasible in clinical studies, the rhythmic expression of clock genes has been examined in peripheral blood mononuclear cells (PBMCs), which found an altered expression of numerous clock genes in OSA patients compared to control subjects [124,125,126]. Although the results obtained from mouse OSA models and human patients are consistent in showing alterations or disturbances in circadian clocks, there are obvious gaps between the two datasets, i.e., tissues/organs vs. PBMC. Given the complex differences in the temporal organization of the circadian system between diurnal and nocturnal species [38], it is hard to predict the extent to which the findings from mouse models can be translated to human patients. Therefore, a diurnal model could be more informative to reveal chronotype-specific circadian phase misalignment in peripheral tissues/cells relative to the SCN.

In addition to altered clock gene expressions, an abnormal melatonin secretion pattern has also been reported. The nocturnal peak of serum melatonin was absent in OSA patients, even after 2 months of continuous positive airway pressure (CPAP) therapy [127,128]. A later study found that the nocturnal peak of melatonin could be restored after CPAP, although it took at least 3 months of treatment [129]. A recent study analyzing the 24 h profile of salivary melatonin in dim light without the forced desynchrony paradigm found a lower amplitude of melatonin rhythm in OSA patients that seemed to correlate with the severity of disease [130]. Besides regulating sleep and circadian rhythms, melatonin also plays important roles in a wide array of physiological and neurological processes due to its anti-oxidative and anti-inflammatory properties [131]. In moderate-to-severe OSA patients, a significant negative correlation was found between serum melatonin with an intestinal barrier function biomarker and inflammatory markers, suggesting that melatonin is likely involved in mediating intestinal barrier dysfunction and systemic inflammation in OSA patients [132]. Therefore, the role of melatonin in OSA-associated pathology could be multifaceted, waiting to be elucidated in animal models. However, the commonly used mouse strain in biomedical research C57BL/6J is melatonin deficient. Although a C57BL/6J line proficient in melatonin synthesis can be genetically engineered [133], the biological property of the hormone is not necessarily the same between diurnal and nocturnal mammals because it is secreted in the sleep/resting phase in one but the awake/active phase in the other. Therefore, a diurnal model with intact melatonin could be beneficial to entangle the relationship between circadian disruption, melatonin, and OSA-associated pathologies.

### 5.3. Smith–Magenis Syndrome

Smith–Magenis syndrome (SMS) is another condition that could benefit from a diurnal animal model. SMS is a neurodevelopmental disorder characterized by intellectual disability, physical health problems, and behavioral problems, with a unique feature of sleep and rhythm disturbances [134,135]. Sleep disturbances such as daytime sleepiness, frequent nighttime awakenings, and decreased total sleep time are experienced by all SMS patients, beginning in early childhood [136]. Most of the patients also display an inverted melatonin rhythm, with higher secretion during the day and lower secretion at night [137,138]. SMS is the only known genetic disorder with an inverted melatonin rhythm [139], which has been postulated as a contributing agent for the sleep problems in SMS [140]. Treatments to restore normal melatonin cycling, such as blocking the daytime rise in melatonin using β1-adrenergic antagonists, melatonin supplementation at night, or the two combined, reportedly alleviate the sleep–wake problems and some of the behavioral issues of SMS patients [141].

SMS is most commonly associated with heterozygous microdeletions of chromosome 17p11, containing 13 genes (~1.5 Mb), of which the retinoic acid-induced 1 (*RAI1*), a chromatin-binding protein, is recognized as the major gene contributing to SMS (reviewed in [137,138,142]). Multiple mouse models of SMS have been developed, which provided important insights into the contributions of RAI1 in SMS-related symptoms, e.g., in feeding/obesity [143]. However, the mouse model (*Rai1*^+/−^ mice) does not show the sleep and rhythm disturbance observed in humans. An earlier study reported generally similar circadian rhythms between *Rai1*^+/−^ and wild-type (WT) mice [144]. A recent study reported that the *Rai1*^+/−^ mice “clearly differ from SMS patients” regarding their sleep and rhythms [145]. Contrary to the reduced sleep and frequent awakening seen in SMS patients [146], *Rai1*^+/−^ mice slept significantly more than WT mice in their resting phase [145]. The striking differences in rhythms and sleep between *Rai1*^+/−^ mice and SMS patients may reflect distinct molecular and neural mechanisms that give rise to the opposite chronotypes between the two species, particularly considering the direct interaction between RAI1 and the molecular clock. It has been found that RAI1 binds to the *CLOCK* gene promoter in human cell lines, and the knockdown of RAI1 in human cell cultures led to decreased expression of several core clock genes, as well as a reduced amplitude and shortened period of the *BMAL1* reporter oscillation [147]. On the other hand, the lack of the sleep and rhythm phenotype in mouse SMS models could also be due to the melatonin deficiency in C57BL/6J mice if the reverted melatonin rhythm in SMS patients is indeed the causal factor for their sleep problems [140]. *Rai1*^+/−^ mice also showed an exaggerated response to light compared to WT, such that their locomotor activity or active waking behaviors were diminished during light exposure [145]. The result indicates that *Rai1* buffers the inhibitory effects of light on locomotor activity and wakefulness in mice; the haploinsufficiency of *Rai1* leads to enhanced inhibition or hypersensitivity to light in *Rai1*^+/−^ mice. However, the effect of *Rai1* in humans might be the opposite of that in mice. In contrast to the hypersensitivity in mice, the haploinsufficiency of *Rai1* in humans likely results in hyposensitivity to light based on the daytime sleepiness and particularly the daytime rise in melatonin observed in SMS patients. Thus, *Rai1*^+/−^ mice failed to recapitulate the sleep rhythm disturbance in SMS patients likely due to (1) intrinsic differences in the circadian time-keeping system between nocturnal and diurnal mammals, (2) melatonin deficiency, and (3) distinct responses to light independent from the circadian drive, which can all be addressed using a diurnal rodent model.

## 6. Conclusions

Model organisms are essential experimental systems for translational research that integrates basic, patient-oriented, and population-based studies with the ultimate goal of improving public health [148]. In the past decades, laboratory mice have become the predominant mammalian model for studying human disorders. Since a mouse genome sequence was published in 2002, a trans-NIH initiative called the Knockout Mouse Project was conceived in 2003 to generate knockout mice that are publicly available to researchers in the biomedical field [149]. The molecular tools available in mice are invaluable for establishing the causal roles of genes in health and disease. However, as discussed in the present review, the chronotype difference, i.e., laboratory mice being nocturnal while humans are diurnal, could present a significant translational flaw when applying the research findings obtained from mice to humans. Although we do not fully understand how the opposite activity patterns arise, the available findings to date suggest that they involve the interplay between the circadian system and the responses to various external or internal factors (e.g., light or melatonin) that can influence sleep or arousal. Thus, the difference between the two chronotypes is much more complex than a simple reversal, such that the physiological or neurological process in diurnal mammals during the day is not equivalent to that in nocturnal ones at night. Although a diurnal model is desirable, especially in conditions when the circadian clock, light, or melatonin are intertwined in the underlying physiology or pathophysiology, a major limitation of diurnal rodents is that they have not been genetically tractable. Previous attempts to generate a germline transgenic line using a diurnal rodent, the Sudanian grass rat, reported repeated failures likely due to “the lack of knowledge of experimental procedures suitable for creating transgenic diurnal rodents” [150]. Recent efforts by our team at Michigan State University have established a set of methods that enable the first successful CRISPR-based genome editing in a diurnal rodent, the Nile grass rat, by targeting the *Rai1* gene [151]. The methods developed include a superovulation protocol, protocols for in vitro embryo culture and manipulation (electroporation or microinjection), and in vivo gene targeting using the improved Genome Editing via Oviductal Nucleic Acids Delivery (i-GONAD) methods. Preliminary data obtained from the newly created *Rai1^+/^*^−^ grass rats showed disturbed daily rhythms and sleep [152], which will help to elucidate the neural mechanisms underlying the sleep rhythm disturbances and melatonin reversal in SMS patients. More broadly, we hope this initial effort will encourage and guide the future development of genetically modified diurnal rodents and their adoption for basic and translational research.
